# A simple optimization can improve the performance of single feature polymorphism detection by Affymetrix expression arrays

**DOI:** 10.1186/1471-2164-11-315

**Published:** 2010-05-20

**Authors:** Youko Horiuchi, Yoshiaki Harushima, Hironori Fujisawa, Takako Mochizuki, Masanori Kawakita, Takayuki Sakaguchi, Nori Kurata

**Affiliations:** 1 Genetic Strains Research Center, National Institute of Genetics, 1111 Yata, Mishima, Shizuoka 411-8540, Japan; 2 Department of Mathematical Analysis and Statistical Inference, The Institute of Statistical Mathematics, 10-3 Midori-cho, Tachikawa, Tokyo 190-8562, Japan; 3 Transdisciplinary Research Integration Center, Research Organization of Information and Systems, Kamiyacho Central Place 2F, 4-3-13 Toranomon, Minatoku, Tokyo 105-0001, Japan; 4 Graduate School of Information Science and Electrical Engineering, Kyushu University, 744 Motooka, Nishi-ku, Fukuoka 819-0395, Japan; 5 Health Informatics and Biostatistics Lab., Oita University of Nursing and Health Sciences, 2944-9 Megusuno, Oita, Oita 870-1201, Japan

## Abstract

**Background:**

High-density oligonucleotide arrays are effective tools for genotyping numerous loci simultaneously. In small genome species (genome size: < ~300 Mb), whole-genome DNA hybridization to expression arrays has been used for various applications. In large genome species, transcript hybridization to expression arrays has been used for genotyping. Although rice is a fully sequenced model plant of medium genome size (~400 Mb), there are a few examples of the use of rice oligonucleotide array as a genotyping tool.

**Results:**

We compared the single feature polymorphism (SFP) detection performance of whole-genome and transcript hybridizations using the Affymetrix GeneChip^® ^Rice Genome Array, using the rice cultivars with full genome sequence, *japonica *cultivar Nipponbare and *indica *cultivar 93-11. Both genomes were surveyed for all probe target sequences. Only completely matched 25-mer single copy probes of the Nipponbare genome were extracted, and SFPs between them and 93-11 sequences were predicted. We investigated optimum conditions for SFP detection in both whole genome and transcript hybridization using differences between perfect match and mismatch probe intensities of non-polymorphic targets, assuming that these differences are representative of those between mismatch and perfect targets. Several statistical methods of SFP detection by whole-genome hybridization were compared under the optimized conditions. Causes of false positives and negatives in SFP detection in both types of hybridization were investigated.

**Conclusions:**

The optimizations allowed a more than 20% increase in true SFP detection in whole-genome hybridization and a large improvement of SFP detection performance in transcript hybridization. Significance analysis of the microarray for log-transformed raw intensities of PM probes gave the best performance in whole genome hybridization, and 22,936 true SFPs were detected with 23.58% false positives by whole genome hybridization. For transcript hybridization, stable SFP detection was achieved for highly expressed genes, and about 3,500 SFPs were detected at a high sensitivity (> 50%) in both shoot and young panicle transcripts. High SFP detection performances of both genome and transcript hybridizations indicated that microarrays of a complex genome (e.g., of *Oryza sativa*) can be effectively utilized for whole genome genotyping to conduct mutant mapping and analysis of quantitative traits such as gene expression levels.

## Background

High-density oligonucleotide arrays are currently the most widely used high-throughput technology for whole-genome gene expression studies. Array technology also makes it possible to genotype thousands of nucleotide polymorphisms (NPs) efficiently [1], and detected NPs are called single feature polymorphisms (SFPs) [2]. In addition to their utility in measuring gene expression levels, whole-genome DNA hybridization to expression arrays has various applications in small genome species; mutant mapping in yeast (genome size: ~12 Mb) and *Arabidopsis *(genome size: ~125 Mb) by bulk segregant analysis [2-7]; quantitative trait loci extreme array mapping in *Arabidopsis *[4,8,9]; and comparative genomics in yeast [10], *Arabidopsis *[11], malaria mosquitoes (genome size: ~278 Mb) [12], in the human malarial parasite (genome size: ~23 Mb) [13], and in *Mycobacterium tuberculosis *(genome size: ~4.4 Mb) [14]. However, in large genome species, such as barley (genome size: ~5.2 Gb) [15], *Xenopus *(genome size: ~3.1 Gb) [16], and maize (genome size: ~2.5 Gb) [17], whole-genome DNA hybridization to expression arrays has not worked out well because of cross-hybridization. Although applications of oligonucleotide expression arrays were limited in large genome species, complementary RNA (cRNA) from their transcripts was used to detect SFPs in barley [15,18,19], maize [17], wheat (genome size: ~17 Gb) [20], and cowpea (genome size: ~600 Mb) [21].

Rice is important as both a food and model plant for the grasses. The genome size of rice (389 Mb) is relatively small amongst crop species, but is larger than that of malaria mosquitoes, which have the largest genome used in successful studies of whole-genome hybridization. Affymetrix supplies a 3'-expression array for rice, the Affymetrix GeneChip^® ^Rice Genome Array (Santa Clara, CA, USA). A trial SFP detection using whole-genome hybridization by the rice array was reported by Kumar *et al*., and more than 70% SFP detection sensitivity at about 10% estimated FDR (False Discovery Rate) was verified by sequencing probe targets [22]. SFP detection using rice transcripts was reported by Kim *et al.*, andthey detected 1,208 SFPs, and 60 out of 62 predicted SFPs were verified by sequencing predicted SFP-containing amplicons [23]. However, because the number of sequenced targets was biased to SFP-predicted ones, it was estimated that the sensitivity would be higher than the true value. Genomes of two rice strains have been fully sequenced. One is *japonica *cultivar Nipponbare, which has been sequenced by a BAC-by-BAC approach [24], and the other is *indica *cultivar 93-11, which has been sequenced by a shotgun approach [25]. Nucleotide differences in coding and 3'-untranslated (UT) regions of genes between the two strains were 3.0 single nucleotide polymorphisms (SNPs)/kb and 4.5 SNPs/kb, respectively [25]. One of the 10 probes was expected to detect an SFP in the 93-11 genome, because each probe was 25-mer long, most rice GeneChip^® ^probes were designed to target the coding and 3'-UT regions of *japonica *transcripts, 4 SNPs/kb were expected in the target region of the 93-11 genome on an average. The two fully sequenced rice strains provided us with an opportunity for detailed analyses of SFP detection efficiency.

First, we searched all probe target sequences in Nipponbare and 93-11 genomes and predicted SFPs between them. Second, we investigated optimized experimental conditions to detect these SFPs by whole-genome hybridization. Several statistical methods for SFP detection were compared using whole-genome hybridization data of Nipponbare and 93-11 cultivars. Effects of several background corrections were also examined for maximum SFP detection performance. Third, SFP detection efficiency by cRNA hybridization was also investigated by applying our recently proposed method for simultaneously detecting nucleotide and expression polymorphisms (SNEP) using the Affymetrix GeneChip^® ^array [26]. Finally, a comparison of benefits and limitations of SFP detection by whole-genome and transcript hybridization approaches was made.

## Results

### BLASTN analysis of Affymetrix GeneChip^® ^array probes

The Affymetrix GeneChip^® ^array was designed for 48,564 *japonica *transcripts and 1,260 *indica *transcripts. A transcript is represented as a probe set. A probe set is made up of several probe pairs (typically 11 pairs) comprised of Perfect Match (PM) and Mismatch (MM) probes. MM probes were designed to represent the non-specific hybridization signal value. Several probes on this array had a possibility of cross-hybridization with other regions of the rice genome because the array probes were designed before the completion of rice genome sequencing efforts. Only 25-mer completely matched and single copy probes of the Nipponbare genome were extracted and used in this study. To select single copy completely matched probes, similarities of all 628,725 PM probe target sequences of the Affymetrix GeneChip^® ^array were searched against the Nipponbare genome by BLASTN analysis (Table 1). A significant number of probes (130,215) had multiple hits on the Nipponbare genome. Some of the probes were designed to splice sites of *japonica *or *indica*-specific transcripts, and 67,531 probes were neither single hits nor were they completely matched to the Nipponbare genome. The remaining 430,979 probes had single hits and complete matches in the Nipponbare genome. We referred to these as unique probes and used them for SFP detection by whole-genome and cRNA hybridizations. By BLASTN analysis of Nipponbare unique probes, 69,255 probes were confirmed to be SFPs against the 93-11 genome. Among the unique probes, 38,009 probes had multiple hits in the 93-11 genome; however, we did not eliminate these as the 93-11 genome has not yet been sequenced. It is hypothesized that SFP detection performance in this study will be similar for unsequenced strains. For SFP detection by cRNA hybridization, we selected 391,818 probes representing 41,525 transcript sets that consisted of more than six unique probes, since SNEP needs several probes in a set of transcripts. Among the 41,525 transcript sets, 58,150 probes out of 391,818 were predicted to be nucleotide polymorphism (NP) containing probes, SFP, for the 93-11 genome.

**Table 1:** Summary of the BLASTN search of probe target sequences on Nipponbare and 93-11 genomes.

	Probe	Probe Set
Total number	628,725	57,194
No hit or single hit but not perfect match to Nipponbare genome	67,531	
Multiple hits to the Nipponbare genome	130,215	
Unique probes in the Nipponbare genome^a^	430,979	51,477
SFP probes for the 93-11 genome in the unique probes for the Nipponbare genome^a^	69,255	22,539

Sets with more than six unique probes in the Nipponbare genome^a^	391,818	41,525
SFP probes to the 93-11 genome in the selected sets^b^	58,150	17,912

^a^, means probe target sequence hit a single site and perfectly match with the Nipponbare genome.

^b^, sets with more than six unique probes in the Nipponbare genome.

### SFP detection by whole genome DNA hybridization

The rice genome, which is about 389 Mb in size [24], may generate much more noise relative to the true signal intensity in genomic DNA (gDNA) hybridization, compared with other organisms with less complex genomes such as those of yeast and *Arabidopsis*. Thus, it is important to investigate conditions that maximize hybridization signal intensity differences between completely matched and NP containing targets of probes on the Affymetrix GeneChip^® ^arrays. There are two types of probes on the Affymetrix GeneChip^® ^arrays; PM and MM. Although the MM probes were designed with single complementary substitutions at the 13th base (midpoint) of each PM probe to represent non-specific hybridization signal values, many studies have indicated that some MM probe intensities are frequently higher than those of the corresponding PM probes (designated: MM > PM) [27,28]. The MM probe sequence is an ideal NP containing a sequence against a complete match target sequence of a PM probe. MM signal intensity would show an ideal SFP intensity for a PM probe. This study thus began as an effort to investigate conditions to maximize the difference between MM and PM intensities for a complete match target.

**Figure 1:**
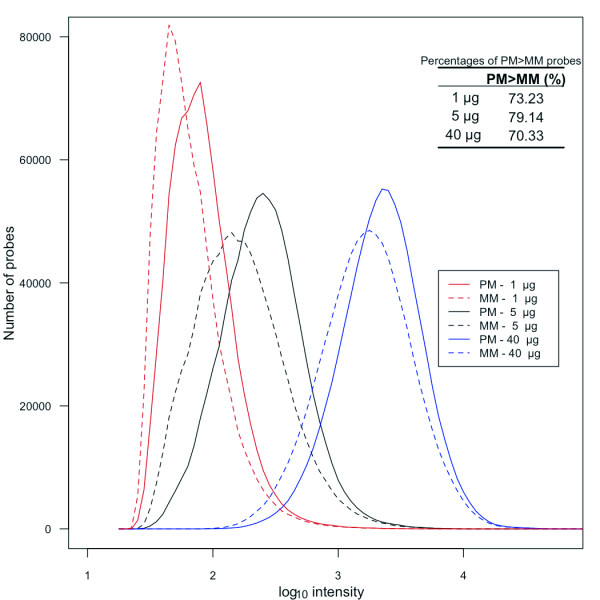
**Effects of applied DNA amounts on signal intensity of PM and MM**. Probe numbers in 0.1 log_10_-intensity windows were plotted with a step width of 0.05 log_10_-intensity. A comparison is shown between the probe number distributions obtained using different quantities of gDNA products.

**Figure 2:**
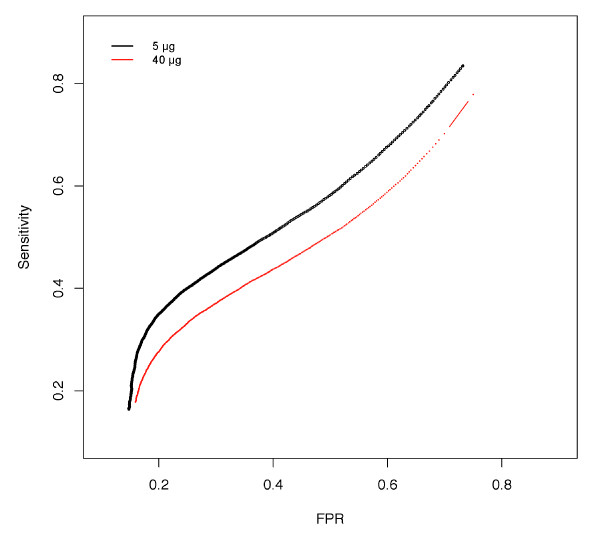
**Improvement of SFP detection performance at the optimized condition**. SFP detection performances were compared by ROC curves. Sensitivity (the ratio of the number of correctly called SFPs to the expected number of SFPs) was plotted against the false-positive rate (the ratio of the number of falsely called SFPs to the total called number of SFPs) by changing the thresholds of analysis.

All Affymetrix protocols for hybridization, washing, and scanning have been standardized [29], and were difficult to change. Amounts of Nipponbare genome DNA products that were randomly amplified and labeled (100-250-bp fragments) were variable on hybridization. ~40 μg of products were obtained in a single labeling reaction, and 1, 5, or 40 μg of reaction products was hybridized. To investigate how the overall intensity distribution of PM and MM changed with increasing amounts of labeled products, distribution of probe numbers of the signal intensities were compared (Figure 1). For the study of density plots, 430,979 unique probes in the Nipponbare genome, chosen by the BLASTN analysis, were used. Although we selected unique probes, both PM and MM probe signals exhibited a wide range of intensities. Signal intensities increased with the increasing amount of products not only for PM probes, but also for MM probes. The increase in MM signal intensities could be due to both non-specific cross-hybridization and binding to target fragments. When 1 μg of the product was applied, the distribution of MM probe intensities appeared to be asymmetric with a lower limit. When 40 μg of product was applied, although the signal intensity was not saturated, the distribution curve of PM signals appeared to be asymmetric. When 5 μg of the product was hybridized, the overall intensity distribution of MM was best separated from that of PM. The number of probes with PM > MM was maximized with 5 μg of product. Consequently, we concluded that ~79% would be the maximum sensitivity for SFP detection by whole-genome hybridization. To evaluate the effects of the optimization on SFP detection, significance analysis of microarray (SAM) [30] was performed on the data of 5 μg and 40 μg applications. SAM has been widely used for SFP detection [2,22,31]. The log_10 _intensities of raw PM values were used to determine SFPs in each analysis. SFP detection performance was evaluated by sensitivity, ratio of the correctly called to the expected number of SFPs, the false-positive rate (FPR), and the ratio of the number of falsely called to total called SFPs. Receiver operating characteristic (ROC) curves with sensitivity plotted against FPR, are shown in Figure 2. In entire threshold, SFP detection in the 5 μg application was better than that in the 40 μg application. The most suitable threshold in the 5 μg application, where the slope of the ROC curve was 1, was at delta = 0.378 for SAM, and the detected number of true SFPs, the sensitivity and FPR were 26,936, 38.89% and 23.58%, respectively (Table 2, Figure 2). In the 40 μg application, the detected number of true SFPs was reduced to 22,030 at a similar FPR (Table 2). The optimization allowed a more than 20% increase in the detected number of true SFPs, although a dramatic increase in intensity of PM > MM probes signals was not observed (from 70% to 79% (Figure 1, inset)).

**Table 2:** SFP detection by gDNA hybridization.

Application^a^	5 μg	40 μg
Called^b^	35,247	28,826

TRUE^c^	26,936	22,030
	38.89%	31.81%

FALSE^d^	8,311	6,796
	23.58%	23.58%

430,979 unique probes in 51,477 probe sets were used for SFP call. ^a^, amount of applied genomic DNA for hybridization. ^b^, number of probes detected as SFP probes at a threshold of delta = 0.378 by SAM. ^c^, number of true SFP calls and percentage of true calls to predicted SFP probes by sequence analysis. ^d^, number of false SFP calls and percentage of false calls to total calls.

**Figure 3:**
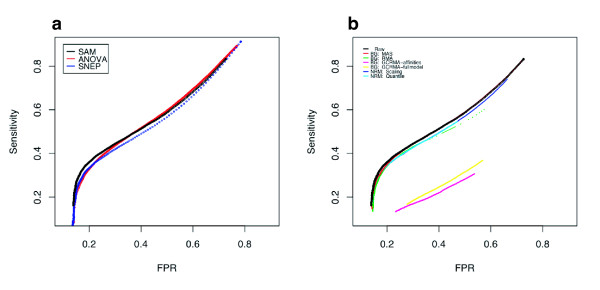
**Comparison of SFP detection performances using different statistical tests and background correlations**. (a) Performances of the three statistical tests for called SFPs: classical Student's *t*-test (SAM, black and ANOVA, red) and a newly developed method for SFP detection (SNEP, blue). (b) The effects of six different signal corrections on SAM analysis: MAS5 (red), RMA (green), GCRMA (their affinity [magenta] and full model [yellow]), and global scaling (dark blue), and quantile normalization (light blue). Definition of "Sensitivity" and "FPR" is the same as in Figure 2.

We compared three methods of SFP detection with the 5 μg application: analysis of variance (ANOVA), SAM [30], and SNEP [26]. ANOVA is a simple and classical test for SFP detection between two strains [1,10-13]. SNEP is our recently developed robust method to detect SFPs as outliers of difference log_10 _PM intensities within a probe set between two strains for cRNA hybridization, and it is also applicable for gDNA hybridization. The log_10 _intensities of raw PM values were used to determine SFPs in each analysis. SFP detection performance was evaluated by ROC curves (Figure 3A). Although the three methods showed similar performances, SAM showed better performance with the stringent threshold where FPR was lower than 0.3. Although both sensitivity and FPR were increased by lowering the threshold level for SFP detection, the FPR reached a limit at about 14% with elevating threshold (Figure 3A). To examine background correction effects, major correlations; MAS5 [32], RMA [27], and GCRMA [33] and normalization methods; scaling [32] and quantile [34] were applied before selection of probes and comparison of their SFP detection performances (Figure 3B). No method had positive effects on the detection of SFP by SAM, and the effects of the corrections with other detection methods were found to be the same (data not shown). As a consequence of above comparisons, a simple SAM of log_10_-transformed PM intensities with no background correction or normalization showed the best performance. The most suitable threshold was at *delta *= 0.378 for SAM, and the sensitivity and FPR were 38.89% and 23.58%, respectively (Table 2, Figure 2 and 3).

**Figure 4:**
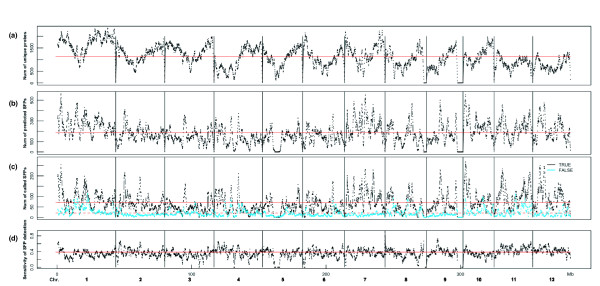
**Distribution of the number of probes in the Nipponbare genome**. Distribution of the number of unique probes (a), predicted SFPs (b), correctly and falsely called SFPs (c), and sensitivity of SFP detection (d) in a 1-Mb window with a step width of 0.1 Mb. SFPs were called by SAM at a threshold of delta = 0.378. TRUE and FALSE are the same as in Table 2. Red lines indicate averages through the genome.

Considering the wide range of potential applications of detected SFPs, it is worthwhile to investigate the distribution of SFPs in the genome. The distribution of the number of unique probes, predicted SFPs, correctly and falsely called SFPs, and the sensitivity of SFP detection in a 1-Mb window are indicated in Figure 4. On an average, each 1-Mb window contained 1,126 probes, and a total of 3,767 windows (covering 382 Mb of the Nipponbare genome) with a step width of 0.1 Mb resulted from a sliding window approach. The distribution of predicted SFPs in the 93-11 genome was similar to the number of synonymous substitutions per site between Nipponbare and 93-11 genes, as reported by Tang *et al*. [35]. However, there were differences in the distribution of SNPs, including in non-coding regions, between Nipponbare and 93-11 genomes [36]. Our correctly called SFP probes were distributed similar to predicted SFP probes and the sensitivity appeared uniformly throughout the genome, although some falsely called probes were observed. This suggests that apart from a small region with high false-positive rates, SFP detection by gDNA hybridization can cover all SNPs, including SNP-poor regions, with considerable accuracy.

### SFP detection using SNEP by rice transcript cRNA hybridization

**Figure 5:**
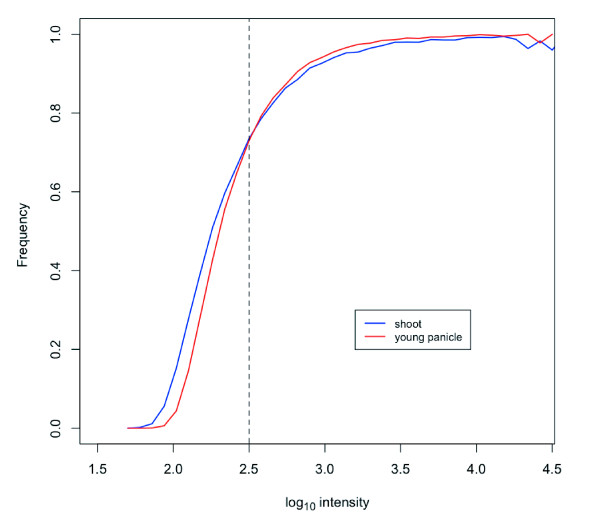
**Effect of signal intensity on the intensity difference between PM and MM probes for completely match transcripts**. Four replicates of shoot or young panicle data were analyzed by ANOVA, and the frequency of probe pairs with significantly different intensities (*p *< 0.05) between PM and MM were plotted against averaged PM signal intensities within 0.1. The dashed line indicates the cut-off signal value (2.5) for mRNA analysis.

**Figure 6:**
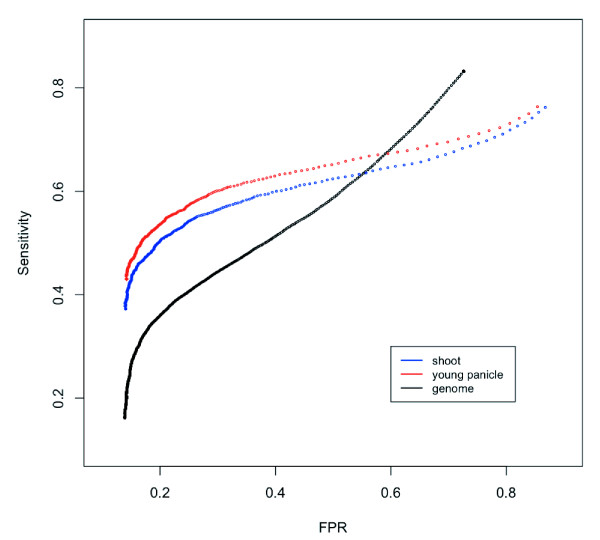
**Comparison of SFP detection performance of transcript and whole-genome hybridizations**. SFP detection performances of shoot (blue) and young panicle (red) transcript hybridizations by SNEP, and of whole-genome hybridization (black) by SAM are represented by ROC curves. Definition of "Sensitivity" and "FPR" is the same as in Figure 2.

Gene expression data from two tissues (shoot and young panicle) of Nipponbare and 93-11 cultivars were obtained according to Affymetrix standard protocols. Raw PM values of all probes were transformed to log_10 _intensities and subjected to SFP calls according to the SNEP procedure [26]. As reported by Fujisawa *et al*., it is difficult to detect SFPs at low expression levels [26]. To investigate the appropriate expression level of SFP detection, PM intensities from 391,818 unique probes for the Nipponbare genome were compared with those for MM by ANOVA (Figure 5). More than 70% of the probe pairs showed significant differences when log_10 _intensities of PM probes were above 2.5 in both the shoot and young panicle. Subsequently, probe sets with a high expression level, where the median log_10 _intensity of a probe set was above 2.5 for both Nipponbare and 93-11 transcripts, were extracted from the unique probe sets. The performance of SNEP using PM intensities for calling SFPs was investigated by ROC curves of the shoot and young panicle (Figure 6). Data of the young panicle showed better performance than the shoot one. This was consistent with results of the comparison between PM and MM intensities of the Nipponbare genome when the log_10 _intensities of PM probe were above 2.5 (Figure 5). SNEP performances at a significance level of 10^-6^, the most suitable threshold on the ROC curve of the shoot (Figure 6), for shoot and young panicle data are summarized in Table 3. SFP detection accuracy with young panicle data was better than that with shoot data, and the number of correctly called SFPs was higher in the young panicle (3,513) than in the shoot (3,393), however, the difference of SFP detection accuracies between young panicle and shoot data was not significant.

**Table 3:** Number of SFPs detected by transcript cRNA hybridization

Tissue type	Shoot	Young panicle	Intersect^a^	Union^b^
Total probe sets	12,620	12,538	11,112	15,768
(Total probes)	(121,763)	(120,708)	(107,129)	(171,482)

SFP probes	6,657	6,549	5,573	7,633

Called	4,324	4,467	3,382	5,409

TRUE	3,393	3,513	2,812	4,094
	51.0%	53.6%	50.5%	53.6%

FALSE	931	954	570	1,315
	21.5%	21.4%	16.9%	24.3%

Genome^c^	2,150	2,208	1,818	2,540

The probe sets with intensity above log_10_-2.5 for both Nipponbare and 93-11 genomes were surveyed. Called indicates the number of probes detected as SFP probes at *p *< 10^-6 ^by SNEP. TRUE and FALSE are same as in Table 2. ^a^, the case applied to the both shoot and young panicle. ^b^, the case applied to either shoot or young panicle. ^c^, the number of SFPs detected by whole-genome hybridization in TRUE.

To investigate effects of selection by gene expression level on SFP detection by transcript hybridization, SNEP analyses were performed for all probe sets. In SNEP analysis, extraction of probes sets does not affect on individual SFP detection performance, because SNEP analysis performs SFP detection on a set by set basis. However, extraction by expression level improved total SFP detection sensitivity and FPR by transcript hybridization (Table 4, Additional file 1). Sensitivities of true SFP detection for shoot and young panicle data at the same SNEP threshold, 10^-6^, were markedly decreased by addition of low expression genes from 51.0% to 6.9% and from 53.6% to 7.7%, respectively (Table 4). FPRs also became worse from 21.5% to 30.2% in shoot and from 21.4% to 27.4% in young panicle. However, in SNEP analyses for all probe sets, increments of the detected numbers of true SFPs in both transcript hybridizations could be achieved without a large increment of false positive SFPs at a stringent threshold, 10^-11.25^~10^-10.05^, (Table 4). SNEP analyses of all probe sets at a stringent threshold are an alternative to those of highly expressed genes at a moderate threshold for obtaining more true SFPs.

**Table 4:** Effects of low expressed gene elimination on SFP detection performances in transcript hybridization by SNEP.

Tissue type	Shoot Expressed	Shoot All		Young panicle All	Young panicle All	
Total probe sets	12,620	41,525		12,538	41,525	
Total probes	121,763	391,818		120,708	391,818	

SFP probes	6,657	58,150		6,549	58,150	

SNEP threshold	10^-6.0^	10^-6.0^	10^-11.25^	10^-6^	10^-6.0^	10^-10.05^

Called	4,324	7,793	5,136	4,467	7,492	5,727

TRUE	3,393	5,442	4,030	3,513	5,440	4,502
	51.0%	9.4%	6.9%	53.6%	9.4%	7.7%

FALSE	931	2,351	1,106	954	2,092	1,225
	21.5%	30.2%	21.5%	21.4%	27.4%	21.4%

Numbers of "Shoot Expressed" and "Young panicle Expressed" were from Table 3. Numbers of SNEP analyses for "all" were summarized both at the same SNEP threshold, 10^-6^, and at the same FPR of "Expressed".

In both shoot and young panicle ROC curves, FPR seemed to have a minimum at around 0.1 (Figure 6). Numbers of falsely called SFPs in shoot and young panicle data were 931 and 954 at a significance level of 10^-6^, respectively (Table 3). Even at a higher threshold, at a significance level of 10^-18^, 447 and 520 falsely called SFPs were observed and 352 probes were common in both the shoot and panicle. Because most of the decrease in intensity of falsely called SFPs appeared to be similar to that of real NPs, we investigated this in detail. One possible cause of a falsely called SFP was gene duplication in the 93-11 genome. In the 266 probe sets with common falsely called SFPs, more than half of these target genes appeared to be multiple copies in the 93-11 genome by BLASTN searching, and false-positive probes did not perfectly hit all multiple targets. Another possible cause of falsely called SFPs is alternative splicing or structural differences between the expressed gene and the model used to design the probes. To confirm the influence of alternative splicing and structural differences on SFP detection, we performed SNEP analysis between Nipponbare shoot and young panicle data, and found a total of 92 falsely called SFPs at a significance level of 10^-18 ^(data not shown). In other words, intron-targeting probes were called as SFPs by SNEP when the expression level of a gene was different. Although 3,393 and 3,513 SFPs were correctly called from shoot and young panicle data, respectively, 2,812 SFPs were identical due to their similar expression profiles (Table 3). A disadvantage of SFP detection by transcript hybridization is that the ability to detect SFPs from RNA data depends on the expression level of a gene in a particular tissue type. To achieve a large number of SFPs, various RNA profiling data from different tissues would be required. In this study, about 40% of correctly called SFPs were detected by transcript hybridization and the remaining were detected by whole-genome hybridization (Table 3).

The distribution of highly expressed genes and correctly called SFPs in the Nipponbare genome was investigated in a manner similar to that of whole-genome hybridization, as indicated in the previous section. The distribution of probes of genes expressed highly in both the shoot and young panicle was the same as that of the unique probes used in this study (Additional file 2). The distribution of correctly called SFPs in both tissues corresponded with that of expressed genes.

### Analysis of false-negative and -positive SFPs by whole-genome hybridization

**Figure 7:**
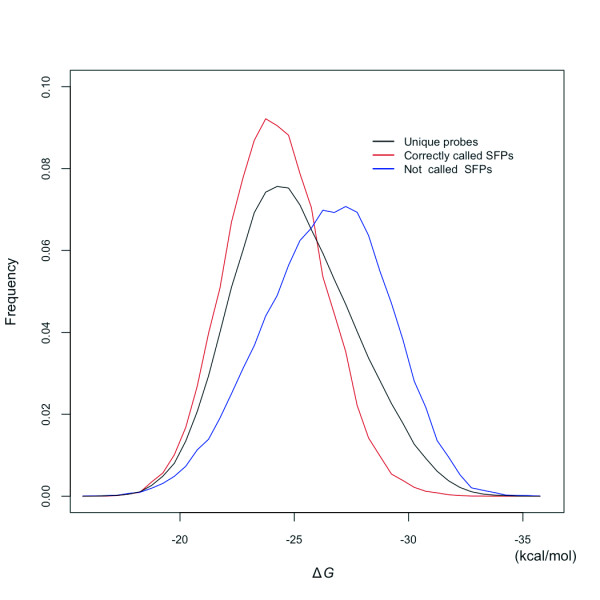
**Effects of probe binding affinity on SFP detection by whole-genome hybridization**. Frequencies of unique probes (black), correctly called SFP probes (red), and false-negative SFP probes (blue) in 0.5 kcal/mol window are plotted against their binding affinities.

SFP detection performance of whole-genome hybridization was inferior to that of transcript hybridization (Figure 6). Low gene expression levels caused low sensitivity in SFP detection by transcript hybridization. Causes of low sensitivity in SFP detection would be different for whole-genome and transcript hybridizations. We examined probe binding affinities. The distribution of binding affinities of all unique probes, correctly called SFP probes, and false-negative SFP probes were investigated (Figure 7). Each PM probe binding affinity (Δ*G*) was calculated by nearest-neighbor thermodynamic parameters [37]. We found that almost all unique probes had binding affinities of approximately -24~-25 kcal/mol, and the plot for correctly called SFP probes indicated a sharp and high peak at around -24 kcal/mol. However, the plot of SFP probes that were not called (false negatives) was shifted to strong binding affinities. This result leads us to conclude that SFP detection by whole-genome hybridization is difficult with probes that have very strong binding affinities. By selecting lower-affinity probes, with Δ*G *> -26 kcal/mol, the SFP detection sensitivity was improved from 38.89% to 56.72% with a similar FPR (24%; data not shown).

**Figure 8:**
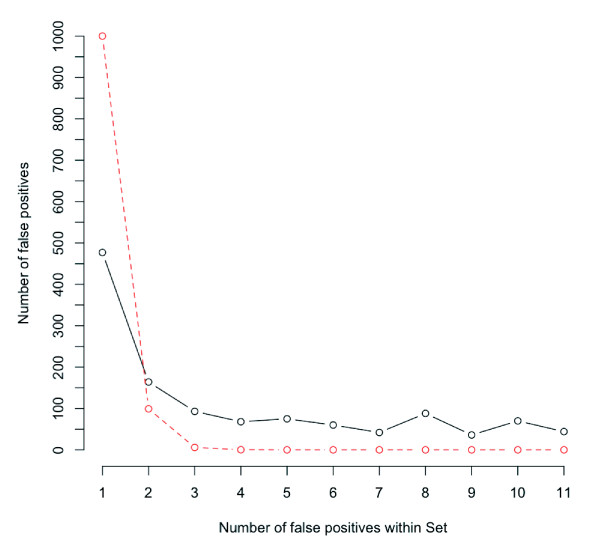
**Distribution of false-positive SFPs in a probe set by whole-genome hybridization**. Falsely called SFPs were added up at every number in a set. Red and black lines show expected and observed value, respectively. Expected values were estimated by a binomial distribution of the false-positive rate.

Even at a stringent threshold of SFP detection, significant false-positive calls existed and there was a minimum FRP limit on the ROC curve at around 0.1 (Figure 2, 3). Sources of the false positives in SFP detection by whole-genome hybridization may be different from those by transcript hybridization. Amplification polymorphisms during whole-genome amplification by annealing random primers to polymorphic sites are possible causes of false positives. Because the amplified fragment length in the case of whole-genome hybridization was 100-250 bp and probes in a probe set were concentrated in a ~300-500 bp region near the 3'-end of genes, false positives by amplification polymorphisms should be clustered. The number of false-positive probes in a probe set were compared with those expected from random occurrences (Figure 8). The observed false-positive probes were not randomly distributed within probe sets and were clustered. This suggested that amplification polymorphisms caused false-positive SFPs by whole-genome hybridization.

## Discussion

Many new applications of oligonucleotide arrays have been developed in recent years. In this study, we describe a method to seek optimal conditions for SFP detection by both genome and mRNA hybridizations using the differences between PM and MM probe signal intensities of completely matched targets. These optimizations greatly improve SFP detection performances in both whole-genome and transcript hybridizations. This simple method is applicable to any other Affymetrix arrays of any species. Especially, for large genome species, this method will be useful to evaluate the possibility of SFP detection.

Under the optimized conditions, SFP detection performances in both genome and mRNA hybridizations were evaluated using the whole genome sequences of the two sequenced strains of *O. sativa *Nipponbare and 93-11. Sensitivity (38.9%) and FPR (22.4%) of SFP detection by whole-genome hybridization was less than the reported sensitivity (57%) and FPR (13%) of SFP detection by genome hybridization of *Arabidopsis *[2]. Following the *Arabidopsis *protocols [31], 40 μg of labeled product was obtained from 300 ng of rice gDNA. The labeled product (40 μg) was used in *Arabidopsis *whole-genome hybridization experiments, and the rice optimal concentration was ~20 times lower than that of *Arabidopsis*, considering their genome sizes. Although a lower target concentration was used for rice whole-genome hybridization and single copy probes were selected for analysis, PM and MM probe intensities could not be sufficiently differentiated. These limitations affected SFP detection. The GC content of rice genes was found to be higher than that of *Arabidopsis *[38,39]. Thus, binding affinities of many rice probes are stronger than those of Arabidopsis, making it more difficult to effectively detect SFPs. In fact, for rice whole-genome hybridization, it was difficult to separate MM probe intensities in the Affymetrix Rice Genome array from PM probe intensities, although overall PM probe intensities were maintained at the same level as those in the GeneChip^® ^*Arabidopsis *ATH1 Genome Array for *Arabidopsis *whole-genome hybridization (data not shown).

Our results from the whole-genome approach suggested that false-positive probes were clustered due to amplification polymorphism caused by nearby SNPs. Thus, these false positives can be used as genetic markers if there is a real NP within 200 bp around them.

Using shoot and young panicle transcripts, higher SFP detection sensitivities (51% and 54%) were observed with a similar FPR of 21%. Comparing our previous results, a sensitivity of 65% and FPR of 10% for 1,901 probe sets from the canonical rice data of the young panicle [26], the SFP detection performances from our present results appears inferior. The differences between previous canonical and present data are as follows: all probe sets of canonical data consisted of 11 probes (in the present case, probe sets with more than 6 probes); all probes in the canonical data set were single copies in both Nipponbare and 93-11 genomes (in the present case, single copy only in the Nipponbare genome); and probe sets that consisted of probes entirely polymorphic to the 93-11 genome were eliminated in the canonical data probe sets (also included in this study). These differences made it difficult for SNEP to detect SFPs, because SNEP detects SFPs as outliers.

Using sequence analysis, in the focused 41,525 probe sets, 17,912 probe sets were expected to have SFP probes in the 93-11 genome (Table 1). However, a substantial number of predicted SFPs were excluded for SNEP analysis of transcripts, because probe intensity with NP was affected only when the gene was expressed at a high level. Even for SFPs containing probe sets with highly expressed gene, 6~7% of sets consisted of more than half the SFP probes and failed to detect SFPs as outliers in a set. This proportion of highly diverse genes was higher than that expected from the random occurrence of mutations. This is not an unusual phenomenon because differences in gene evolution rates are often observed. Most NPs in highly divergent genes were undetected by SNEP because of the difficulty in distinguishing outliers of log_10_-intensity differences in a set. On the other hand, several issues aside from NP, such as alternative splicing or gene duplication, lead to SFPs. Gene duplication can result from unequal crossing over in chromosomal duplication, the outcomes of which can be quite different. Difference in exon-intron structure between duplicated genes in the 93-11 genome could also lead to these misclassifications. Some false positive SFPs detected by transcript hybridizations were attributed to various forms of genetic diversity such as copy number of a gene and alternative splicing. In other words, SNEP detects differences not only in nucleotide sequences and expression levels of a gene, but also in the gross structure of a gene.

More than 27,000 SFPs could be detected by whole-genome hybridization, and about 3,500 SFPs and precise expression polymorphisms could be simultaneously detected by SNEP using transcript hybridization. A technology for simultaneous genotyping by polymorphic markers densely covering the whole genome can be utilized for various applications. Analysis of gene expression levels as a quantitative trait (expression Quantitative Trait Locus: eQTL) is a promising application of SFPs. In eQTL studies of yeast, genotypes of segregants were determined by about 3,000 SFPs from gDNA hybridization, and gene expression levels were determined by another type of microarray with spotted PCR products of the genome [40-42]. The first global eQTL study in a plant was performed in Arabidopsis using 211 recombinant inbred lines, in which genotypes of 540 SFP markers and gene expression levels were evaluated by Affymetrix expression arrays [43]. In barley, using Affymetrix expression arrays and 139 doubled haploid lines, more than 2,000 genetic markers were identified and underwent eQTL analysis with 512 unique segregation patterns in the population [44]. Because these eQTL studies used experimental populations with a limited number of recombinations, the number of genetic markers required for eQTL analysis was not so large. To perform eQTL in rice using an experimental population with homozygous genotypes between *japonica *and *indica*, application of SNEP using transcript hybridization would provide a sufficient number of genetic markers and a robust estimation of gene expression levels.

Recent revolutionary developments in sequencing technologies have challenged microarray technologies [45,46]. However, the Affymetrix GeneChip^® ^array analysis by bulk gDNA hybridization is a cost-effective option for mapping a gene by bulk segregant analysis or QTL extreme mapping, because the rice genome is more than 2.5 times larger than that of Arabidopsis and the required number of reads by a sequencer should be proportional to the genome size.

## Conclusions

In this study, we describe a method to seek optimal conditions for SFP detection by both genome and mRNA hybridizations using the differences between PM and MM probe signal intensities of completely matched targets. The optimizations allowed a more than 20% increase in true SFP detection in whole-genome hybridization and a large improvement of SFP detection performance in transcript hybridization. Significance analysis of the microarray for log-transformed raw intensities of PM probes gave the best performance in whole genome hybridization, and 22,936 true SFPs were detected with 23.58% false positives by whole genome hybridization. For transcript hybridization, stable SFP detection was achieved for highly expressed genes, and about 3,500 SFPs were detected at a high sensitivity (> 50%) in both shoot and young panicle transcripts. High SFP detection performances of both genome and transcript hybridizations indicated that microarrays of a complex genome (e.g., of *Oryza sativa*) can be effectively utilized for whole genome genotyping to conduct mutant mapping and analysis of quantitative traits such as gene expression levels.

## Methods

### BLASTN analysis of Affymetrix GeneChip^® ^array probes

To detect all SFPs with the Affymetrix GeneChip^® ^array in Nipponbare and 93-11 genomes, target sequences for all 628,725 PM probes were searched in both genomes using BLASTN version 2.2.8 [47] (total 371 Mb Nipponbare genome from GenBank/EMBL/DDBJ accession: AP008207 to AP008218 and total 479 Mb 93-11 genome including unmapped contigs of 105 Mb from version 2003-08-01 BGI) under the following conditions: expectation value was 20, match score was 1, mismatch score was -3, cost to open a gap open was 5, and cost to extend a gap was 2. The score of a complete match to the probe target sequence is 25. The score of a single mismatch, depending on the mismatch position, between the target sequence and corresponding probe is from 24 to 21. When the mismatch is in the distal three bases, BLASTN counts a continuous match; however, at inner positions, the score is 21. The score of a single insertion in the target sequence is from 24 to 18, in the same manner. We summarized BLASTN search results of scores greater than or equal to 18. Using this search, if a probe sequence hit only a single region in the genome we considered the probe to be present as a single copy in the genome.

### DNA/RNA preparation and microarray experiments

gDNA from leaves of two rice subspecies, *O. sativa *L. ssp. *japonica *cv. Nipponbare and ssp. *indica *cv. 93-11, was isolated using the Qiagen DNeasy Plant Mini Kit (QIAGEN GmbH, Hilden, Germany). Purified DNA (300 ng) was labeled according to Arabidopsis Protocols [31], and reaction products were hybridized to the Affymetrix GeneChip^® ^arrays according to the Affymetrix standard protocol for RNA [28]. Total RNA was extracted from 3~4-week old shoots of both Nipponbare and 93-11 cultivars using the QIAGEN RNeasy Plant Mini Kit according to the manufacturer's protocol. Labeled cRNA was prepared and hybridized to the Affymetrix GeneChip^® ^arrays according to the manufacturer's guidelines [29]. The Affymetrix GeneChip^® ^arrays were scanned with an Affymetrix GeneChip^® ^Scanner 3000, and raw CEL files were generated by the Affymetrix GeneChip^® ^Operating Software version 1.3. To investigate SFP detection performances of the two rice cultivars, four and five biological replicates of the hybridization as well as data read were carried out for independent gDNA and transcript samples, respectively. These array data were submitted to the Gene Expression Omnibus at http://www.ncbi.nlm.nih.gov/geo/, GSE16341. Gene expression data from 2-cm long young panicles of the two rice subspecies could be obtained as GSE16265.

### Statistical analysis of SFPs using array data

For SFP detection by whole-genome hybridization, all statistical analyses were performed with the freely available statistical package R. The raw CEL intensity files were analyzed using a series of methods implemented by the software Bioconductor [48]. Background correction and normalization algorithms are available in the affy and gcrma packages. The log_10_-transformed intensity value of each feature was extracted and subjected to data analysis for SFP calls using ANOVA, SAM in package "siggenes" [30], and SNEP http://www.ism.ac.jp/~fujisawa/SNEP/. SNEP was originally developed for transcript hybridization; however, it can also be used for SFP detection by whole-genome hybridization after modification of the SNEP script. PM probe intensities were transformed to log10 values; these are randomly grouped together in 500 PM probes, and SFPs were called in each group. This script is available at the SNEP site. SFP detection by transcript hybridization was performed using SNEP, as described previously [26].

### Calculation of probe binding affinity on the Affymetrix GeneChip^® ^array

The binding stability (Δ*G*) of a PM probe to a complete match target at a wash temperature of 50°C was calculated according to the values of the nearest-neighbor thermodynamic parameters for DNA [37]. The binding stability was used for analysis of false-negative SFPs.

## Authors' contributions

YH carried out the experiments, analyzed the microarray data, and prepared the paper. YH conceived of the study, participated in its design, analyzed sequence data, and edited the manuscript. HF modified SNEP and analyzed the microarray data. TM analyzed sequence data. MK analyzed the microarray data. TS modified SNEP. NK conceived of the study, participated in its design, edited the manuscript, and coordinated this study. All authors read and approved the final manuscript.

## Supplementary Material

Additional file 1 **Effects of set extraction by their gene expression level on SFP detection performance**. SFP detection performances between highly expressed genes and all genes were compared by ROC curves. Click here for file

Additional file 2 **The distributions of numbers of the designed probes, ones with highly expression, and the sensitivity of SFP detection across Nipponbare genome**. The distributions of numbers of the designed probes (top), ones with highly expression (middle), and the sensitivity of SFP detection at p < 10^-6 ^by SNEP (bottom) across Nipponbare genome in each 3-Mb segment. Click here for file
