# Supplementary material for: A simple optimization can improve the performance of single feature polymorphism detection by Affymetrix expression arrays
Source: BMC Genomics. 2010 May 20;11:315. doi: 10.1186/1471-2164-11-315 (PMC2885369; doi:10.1186/1471-2164-11-315)
Supplement: Additional file 1 — Effects of set extraction by their gene expression level on SFP detection performance. SFP detection performances between highly expressed genes and all genes were compared by ROC curves. [file 1471-2164-11-315-S1.PDF]

Additional file 1 Effects of set extraction by their gene expression level on SFP detection performance.

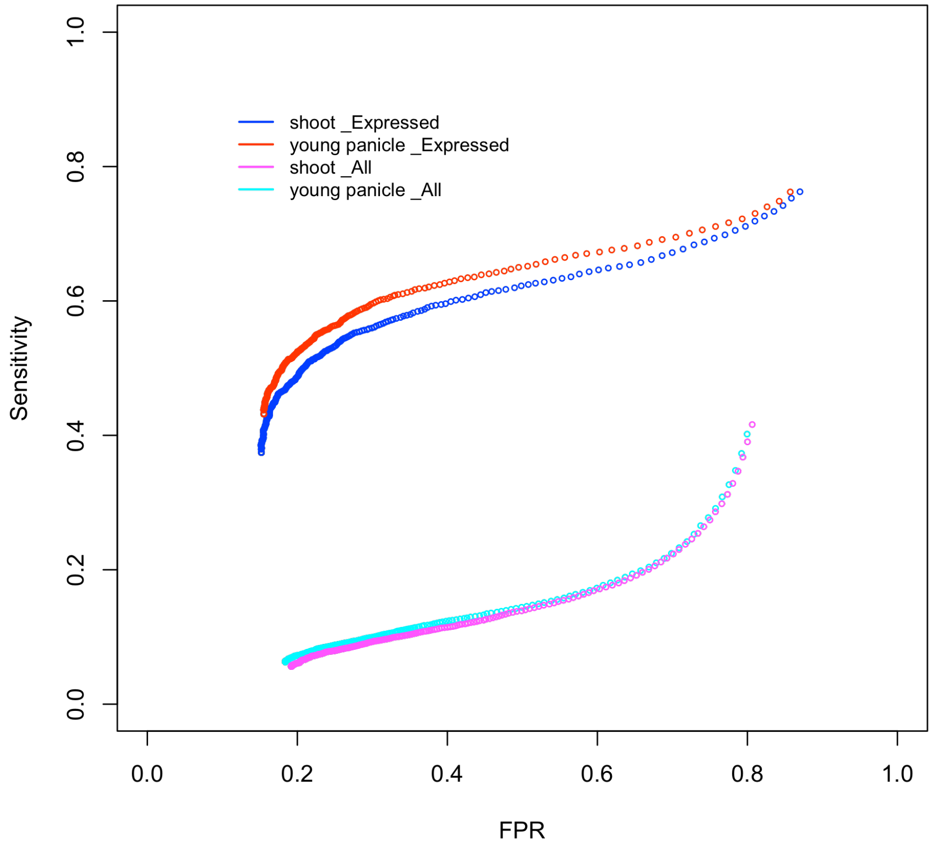

SFP detection performances are represented by ROC curves. SNEP analyses were perfomed only for highly expressed genes, where the median $\log_{10}$ intensity of a probe set was above 2.5 for both Nipponbare and 93-11 transcripts in shoot (shoot_Expressed), and in young panicle (young panicle_Expressed). “shoot_All” and “young panicle_All” show results of SNEP analyses of 41,525 probe sets (Table 1) in shoot and young panicle transtript hybridizations, respectively.
