# Supplementary material for: A simple optimization can improve the performance of single feature polymorphism detection by Affymetrix expression arrays
Source: BMC Genomics. 2010 May 20;11:315. doi: 10.1186/1471-2164-11-315 (PMC2885369; doi:10.1186/1471-2164-11-315)

Additional file 2. The distributions of numbers of the designed probes (top), ones with highly expression (middle), and the sensitivity of SFP detection at $p<10^{-6}$ by SNEP (bottom) across Nipponbare genome in each 3-Mb segment. The step size is 0.3 Mb. Red line in the top panel shows the average through genome. Red and blue lines in the middle and bottom panel indicate the averages throughout genome for young panicle and shoot, respectively.

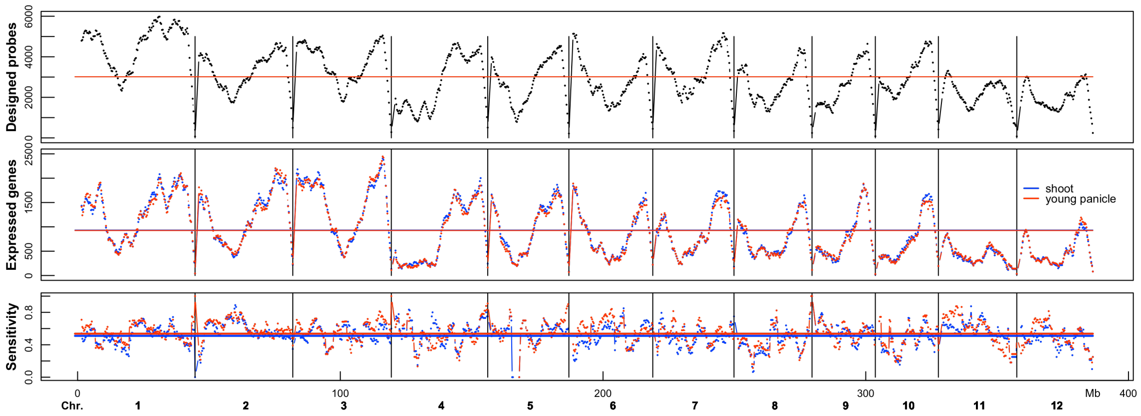

Supplement: Additional file 2 — The distributions of numbers of the designed probes, ones with highly expression, and the sensitivity of SFP detection across Nipponbare genome. The distributions of numbers of the designed probes (top), ones with highly expression (middle), and the sensitivity of SFP detection at p < 10-6 by SNEP (bottom) across Nipponbare genome in each 3-Mb segment. [file 1471-2164-11-315-S2.PDF]
